# Predicting the relative impacts of maternal and neonatal respiratory syncytial virus (RSV) vaccine target product profiles: A consensus modelling approach

**DOI:** 10.1016/j.vaccine.2016.10.073

**Published:** 2017-01-05

**Authors:** Wirichada Pan-Ngum, Timothy Kinyanjui, Moses Kiti, Sylvia Taylor, Jean-François Toussaint, Sompob Saralamba, Thierry Van Effelterre, D. James Nokes, Lisa J. White

**Affiliations:** a Mathematical and Economics Modelling (MAEMOD) Research Group, Mahidol-Oxford Tropical Medicine Research Unit (MORU), Faculty of Tropical Medicine, Mahidol University, Bangkok, Thailand; b Department of Tropical Hygiene, Faculty of Tropical Medicine, Mahidol University, Bangkok, Thailand; c School of Mathematics, Alan Turing Building, University of Manchester, Oxford Road, Manchester, UK; d KEMRI-Wellcome Trust Research Programme, KEMRI Centre for Geographic Medicine Research – Coast, Kilifi, Kenya; e GSK Vaccines, Wavre, Belgium; f School of Life Sciences and WIDER, University of Warwick, Coventry, UK; g Nuffield Department of Medicine, University of Oxford, Oxford, UK

**Keywords:** Transmission model, RSV, Kenya, Vaccine TPP, Hospitalization, Contact pattern

## Abstract

**Background:**

Respiratory syncytial virus (RSV) is the major viral cause of infant and childhood lower respiratory tract disease worldwide. Defining the optimal target product profile (TPP) is complicated due to a wide range of possible vaccine properties, modalities and an incomplete understanding of the mechanism of natural immunity. We report consensus population level impact projections based on two mathematical models applied to a low income setting.

**Method:**

Two structurally distinct age-specific deterministic compartmental models reflecting uncertainty associated with the natural history of infection and the mechanism by which immunity is acquired and lost were constructed. A wide range of vaccine TPPs were explored including dosing regime and uptake, and effects in the vaccinated individual on infectiousness, susceptibility, duration of protection, disease severity and interaction with maternal antibodies and natural induced immunity. These were combined with a range of vaccine implementation strategies, targeting the highest priority age group and calibrated using hospitalization data from Kilifi County Hospital, Kenya.

**Findings:**

Both models were able to reproduce the data. The impact predicted by the two models was qualitatively similar across the range of TPPs, although one model consistently predicted higher impact than the other. For a proposed realistic range of scenarios of TPP combinations, the models predicted up to 70% reduction in hospitalizations in children under five years old. Vaccine designs which reduced the duration and infectiousness of infection were predicted to have higher impacts. The models were sensitive to the coverage and rate of loss of vaccine protection but not to the interaction between vaccine and maternal/naturally acquired immunity.

**Conclusion:**

The results suggest that vaccine properties leading to reduced virus circulation by lessening the duration and infectiousness of infection upon challenge are of major importance in population RSV disease control. These features should be a focus for vaccine development.

## 1 Background

A major burden of respiratory syncytial virus (RSV) arises from infection in the first year of life, particularly the first 3–6 months of life where resultant disease is most severe, most hospitalizations occur and mortality is highest [1]. There are an estimated 3 million cases of severe lower respiratory tract infection and up to 200,000 deaths in children under five years of age per year attributable to RSV [1]. While RSV disease is globally important, the greatest share of the childhood burden is found in the developing world [1]. Hence, while vaccines are needed for both developing and developed countries, we focus in this paper on the low resource setting. The RSV vaccine pipeline is healthy, with over 60 vaccines under development, and whilst most are at pre-clinical or early clinical stages, two are in phase 2 trials and one in phase 3 [2].

In this context, we undertook to model the potential impact of vaccination against RSV infection and disease with respect to the possible vaccine target product profiles (TPPs) and delivery options, and specifically in relation to reduction in early childhood hospitalization. This gives rise to some challenges including the unpredictable response of vaccine due to immature immunity of infants and interaction with maternally derived specific antibodies. Further challenges arise from uncertainties in the mechanisms of acquisition and waning of immunity and the natural history of RSV. Specifically, there is poor understanding of the relationship between susceptibility to RSV infection and repeated exposure. If, for instance, vaccination leads to a reduction in the rate of infection with RSV, how would that impact on the immunity or susceptibility population profile? Different scenarios of waning immunity lead to different modelling structures [3], [4]. Whereas models frequently address uncertainty in the form of sensitivity analyses, in few instances is structural uncertainty investigated [5], [6], [7]. As a consequence, in this study, two structurally distinct mathematical models of RSV were constructed independently, from which to identify consensus predictions: although the consensus modelling approach has been explored for RSV previously [8], [9], it is the first time to include full age-structure and to be used in the context of RSV vaccination. The findings should inform the potential individual and population-level benefits of defined vaccine properties, to anticipate possible limitations in vaccine designs, and galvanize discussion among various vaccine stakeholders early in a vaccine’s development.

## 2 Materials and methods

### 2.1 Data

Data sets from coastal Kenya were used in the modelling exercise representative of the epidemiology of RSV in the low income setting. These data define population demographic structure, age-specific contact rates and age- and time-related RSV diagnosed hospitalization data.

### 2.2 Kenya demographic data

The age-specific fertility and mortality rates used in the model were obtained from the registers of the Kilifi Health and Demographic Surveillance System (KHDSS) for the mid-year estimates for 2007. For more information on the KHDSS, please refer to Scott et al. [10] (see the Supplementary file 3E).

### 2.3 Kenya age-specific contact rates

Diary contact data from a study conducted in the Kilifi KHDSS [11] were used to construct a matrix of age-specific daily rates of contacts with different individuals from which to estimate a ‘Who Acquires Infection From Whom’ (WAIFW) matrix that is central to the age-related transmission compartmental models [12]. The method has been described elsewhere [4] (see the Supplementary file 3E).

### 2.4 Kenya disease surveillance dataset used to optimise the model pre-vaccination

We used numbers of laboratory diagnosed RSV paediatric severe or very severe pneumonia hospital admissions stratified by age and time from paediatric admissions surveillance to Kilifi County (formally District) Hospital coastal Kenya from Oct 2004 to Dec 2010 [13]. Monthly case reports stratified by the monthly age classes for the first two years of life and then yearly from age 24 to 59 months. Each model was fitted to (i) monthly admissions pooled across all age classes and (ii) average numbers of admissions over the time period by age class.

### 2.5 Ethics statement

Approval for using the secondary data from Kenya was obtained from the KEMRI-Wellcome Trust Research Programme’s Data Governance Committee.

### 2.6 Models

Two age-stratified deterministic compartmental mathematical models for simulating the transmission dynamics of RSV were developed. Each had similar layers of structure, namely, demographic, epidemiological, seasonality, disease related and vaccine design and administration. The two models differed, however, in epidemiological structure reflecting uncertainty associated with the natural history of infection and particularly with the mechanism by which immunity was acquired and lost. The key difference between the two models was that one models sequential infections leading to acquisition of a partially immune state that was lifelong (SAI model) [4], whereas the other model assumed that a partially immune state was maintained only by repeated or boosting infections and wanes in the absence of challenge (BWI model) [3]. In other ways the models were harmonised with respect to the model parameterisation and calibration to allow reasonable comparison of output. Full details of each of the two models and the standard technique for model fitting are to be found in the Supplementary materials.

### 2.7 Vaccination framework

**Fig. 1:**
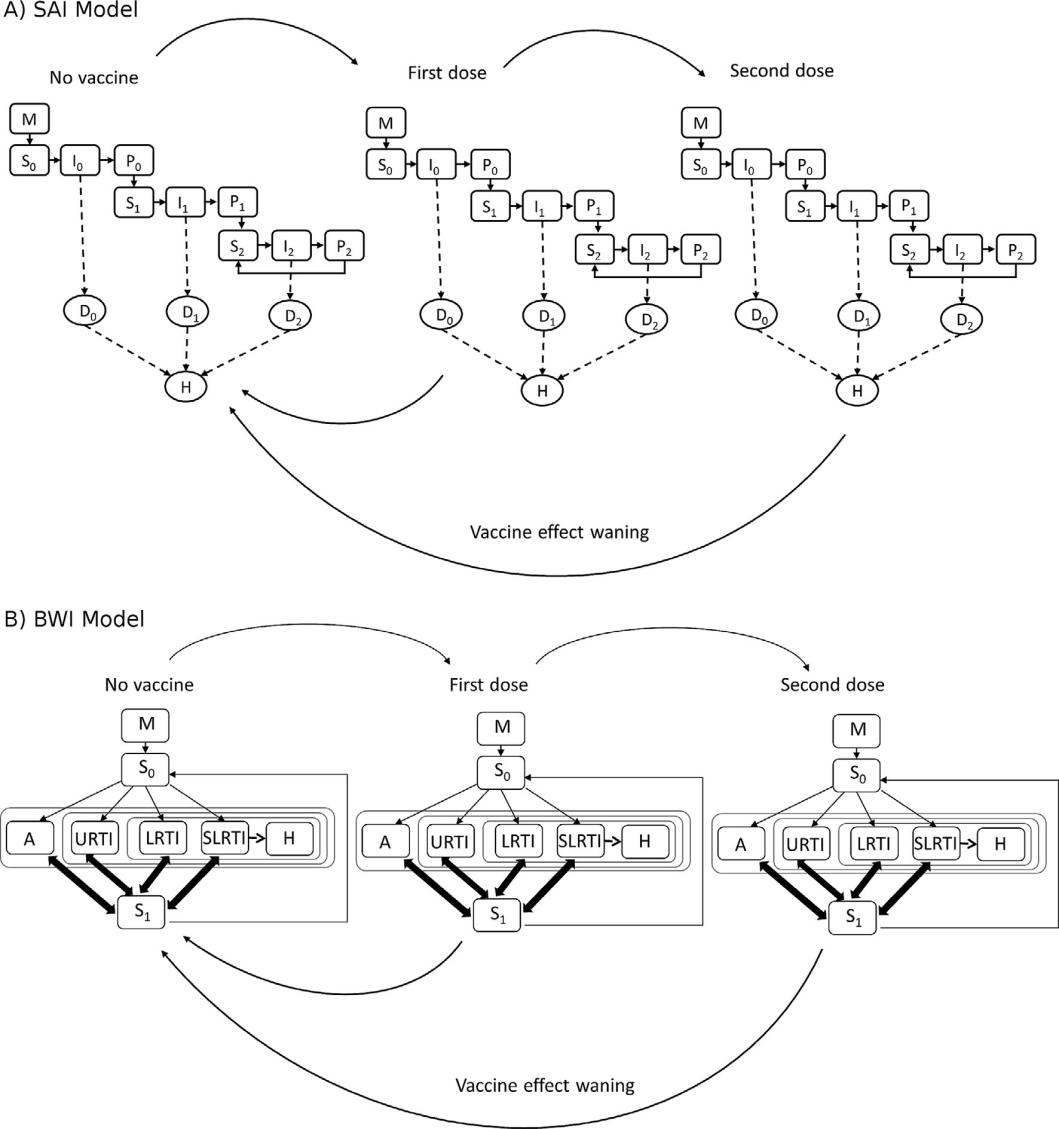
Schema for vaccination implementation in (A) SAI model and (B) BWI model. Arrows show the direction of individuals on receipt of vaccine (moving right) or on loss of vaccine effects due to waning immunity (moving left).

A vaccination framework was developed to interact with the epidemiological sub-model and facilitate the evaluation of vaccines TPPs and delivery strategies. A projection of the population-level impact of vaccination has to account for the uncertainties about the effectiveness of the future vaccines, in particular the types of effects of the vaccine such as: (i) reduction in risk of infection, (ii) reduction in the duration or (iii) the magnitude of viral shedding, (iv) reduction in the risk of upper respiratory tract infection (URTI), (v) reduction in the risk of lower respiratory tract infection (LRTI) or (vi) severe lower respiratory tract infection (SLRTI). There are also uncertainties about the duration of vaccine protection, the natural history of RSV to be accounted for, in particular how the natural immunity builds up after the first and subsequent RSV infections and the interactions of these vaccines with natural immunities i.e. maternal and from prior natural exposures. We explored a wide range of TPPs which are detailed in the following section. Fig. 1 shows vaccine implementation in the SAI and BWI models up to two doses for simplicity.

### 2.8 Vaccine target product profiles (TPPs)

We divide the vaccine TPP into the seven components described as follows. In all instances below we show the baseline choices in **bold** type.

#### 2.8.1 Vaccine effects

The vaccine candidates can potentially have any combination of the following effects on vaccinated individuals in relation to risk of infection and outcomes following infection while the baseline is the combination of all baseline choices in all effects (see Table 1).

#### 2.8.2 Dosing regimen

Infant vaccination was assumed to be either through two or three doses and at various ages of delivery as follow:

(i) 2 doses at 0 and 2 months of age
(ii) **2 doses at 2 and 4 months of age (baseline choice)**
(iii) 3 doses at 0, 1 and 2 months of age
(iv) 3 doses at 2, 4 and 6 months of age

For the SAI model routine (universal) vaccination is implemented as individuals pass through an age–gateway, e.g. for vaccination at age 2 months, a proportion of individuals are vaccinated as they transition out of age 1 month based on the demographic schedule. For the BWI model a vaccination rate is applied to individuals as they pass the age-gateway such that a proportion equal to the required final coverage is attained within a short time period (3 days after the vaccination age). Numerically, this represents a minor difference between the two models (see the Supplementary file 3C).

#### 2.8.3 Waning vaccine effect

Two options for average duration of vaccine effect are **1 year (baseline choice)** and 2 years. For the SAI model the rate of flow from Dose n to Dose (n − 1) is υ i.e. 1/υ is the average duration of effect of each dose, and for the BWI the rate of flow from Dose n to Dose 0 is υ i.e. 1/υ is the average duration of vaccine effect irrespective of number of doses.

#### 2.8.4 Coverage and compliance combinations

We assumed a per dose compliance of either 90% or **100% (baseline choice)**. Poor compliance was applied only when the final coverage will allow this assumption. For example a final coverage of 90% would not allow compliance at 90% for more than one dose because two doses at 90% compliance would imply that the first dose coverage must exceed 100%. The designated final coverage levels were 50%, 70% and **90% (baseline choice)**.

#### 2.8.5 Interaction with maternal antibodies

A range of scenarios are plausible for the potential interaction of an infant vaccine in the presence of a maternal antibody presence that is waning. We mainly focused on three options which were: (i) **“No interaction” (baseline choice)**, (ii) “Bounce up” and (iii) “Drop back” (see the Supplementary file 3A).

#### 2.8.6 Interaction with natural immunity

Three options of effects of vaccination of individuals with natural immunity were explored: (i) **“No effect” (baseline choice)**, (ii) “Multiplicative” and (iii) “Top-up” (see the Supplementary file 3B).

#### 2.8.7 Maternal vaccination

We assume that if children are born to mothers who received maternal vaccination they would be similarly affected as if vaccinated as infants but with the protection commencing from birth and lasting for either **3 months (baseline choice)** or 6 months. The final vaccine coverage considered here are 25%, **50% (baseline choice)** and 75%. Implementation is identical to the process of infant vaccination for each model with a per dose compliance of either 90% or **100% (baseline choice)** (see the Supplementary file 3D).

### 2.9 Sensitivity analysis

We approach the sensitivity analysis in two stages, one-way and multi-way analyses (see the Supplementary file 1).

## 3 Results

**Fig. 2:**
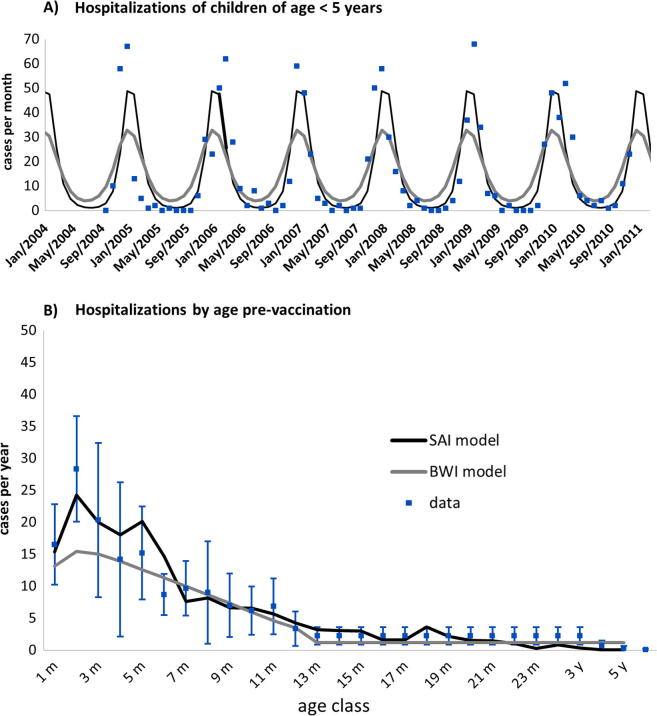
Calibrated fits of the two RSV models for the Kenya setting (black line, SAI; grey line, BWI). (A) Models calibrated using Kilifi, Kenya RSV hospital surveillance monthly time series 2004–2011 (blue markers, upper panel) and (B) the average profile (blue markers and error bars for 95% CI) for annual hospitalized cases stratified by monthly age classes.

Both models could demonstrate reasonable fitting to the Kenya hospitalization data by time and age classes (see Fig. 2).

**Fig. 3:**
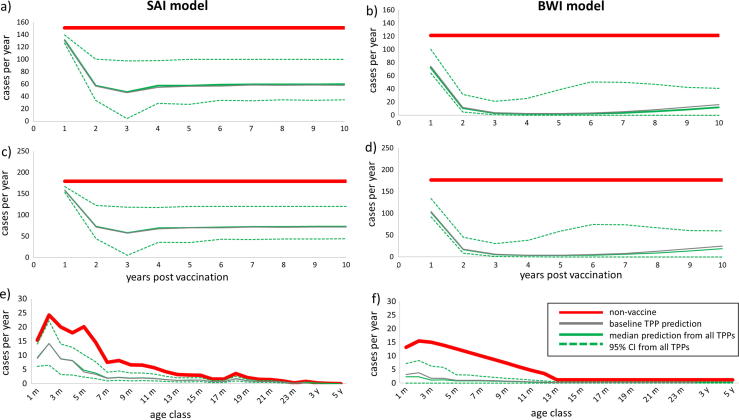
Comparison of predicted impact of routine infant RSV vaccination using two models, SAI model (left column) and BWI model (right column) i.e. predictions of impact of vaccine TPPs on hospitalizations of: infants under 1 year old over time since vaccination begins (a and b); children under 5 years old over time since vaccination begins (c and d); age profiles 10 years after vaccination begins (e and f). Each graph plots the non-vaccine model fit (bold solid red line), the median prediction from all TPPs (solid green line), the 95% prediction limits from all TPPs (dashed green line) and the baseline TPP prediction (bold solid grey line).

**Fig. 4:**
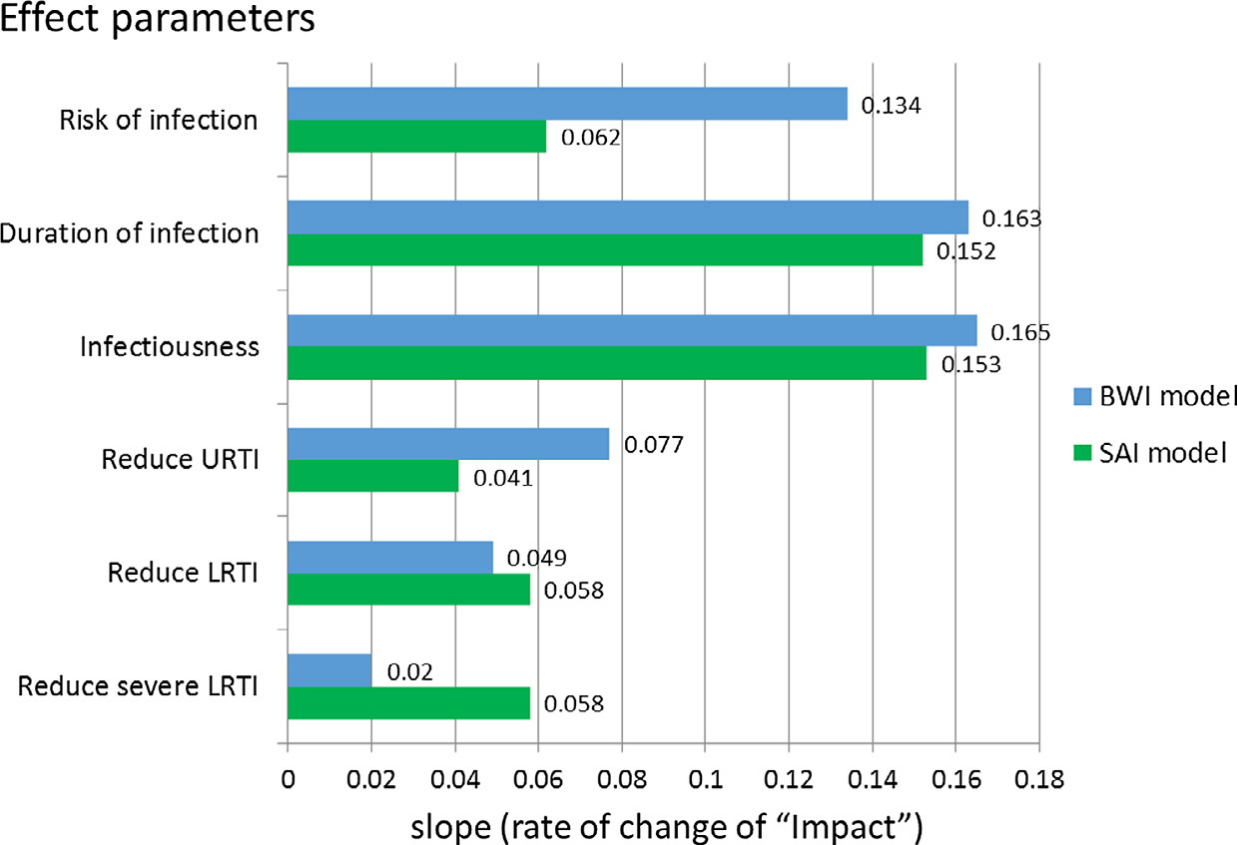
Comparison of change in impact arising from changes in any one of the six ‘effect’ properties of a RSV vaccine (green for SAI; blue for BWI). Univariate regression of impact (percentage reduction in under 5 hospitalization) against the slope of the regression lines which measured the rate of change of impact for each of six ‘effect’ characteristics.

The main outcome impact prediction of the models is the percentage reduction in the number of under 1 and 5 year old hospital admissions after first 10 years of the vaccination programme when compared to the pre-vaccination average. The highlights of the projections of the impact of vaccine TPPs using the two model structures (SAI and BWI) are described in Fig. 3, Fig. 4.

SAI model (left column) and BWI model (right column) predictions of impact of vaccine TPPs on annual hospitalizations of (a) infants under 1 year old over time since vaccination begins (top row); (b) children under 5 years old over time since vaccination begins (middle row); (c) age profiles 10 years after the vaccination programme begins (bottom row). Each graph plots the no-vaccine model fit (bold solid red line), the median prediction from all TPPs (solid green line), the 95% prediction limits from all TPPs (dashed green line) and the baseline TPP prediction (solid grey line).

From Fig. 3, it can be seen that the median impact (solid green lines), which is very similar to that for the baseline parameter set (solid grey lines), is a 50% reduction or more within a year of two of vaccine introduction. The impact tends to be highest early into the vaccine era, with a degree of rebound 5–6 years following vaccine introduction. The projected impact is greater for the BWI model than for the SAI, although the latter is more stable over time. The projected impact by age is in proportion to the pre-vaccine age-related admission proportion.

The key difference between the models is the magnitude of the projected impact. This is a result of the force of infection required to fit the SAI model being higher than that for the BWI model (see the Supplementary file 2 for the model parameter estimates). A preliminary exploration of both models indicated that increasing the force of infection in the BWI model will reduce the predicted impact and that reducing the force of infection in the SAI model will increase the predicted impact (results not shown).

Analyses of the degree of influence of specific TPP component characteristics on the impact of vaccination are summarised in Fig. 4. Specifically, this explores the relative benefits of the different possible vaccine ‘effects’ (see Section 2.8.1.). These results record the univariate linear regression coefficients of change in impact, defined as percentage reduction in under 5 year olds hospitalized, as the level of effect changes (e.g. percent reduction in risk of infection afforded by the vaccine). The outcome measure is thus the degree of influence of a change of effect (of a particular type) on the reduction in the under 5 year old hospitalization. For example, keeping other factors constant, for each 1 unit (i.e. 1%) increase in vaccine effect on risk of infection, we expect a 0.062% and 0.134% reduction in the under 5 year old hospitalization for SAI and BWI model, respectively.

Fig. 4 clearly indicates the relatively important additional benefits of a vaccine that would be able to reduce infectiousness and/or the duration of infection upon RSV-related hospitalization in <5 year old. Other vaccine effects are less influential, i.e. changes in per person risk of infection or of disease arising following infection (whether URTI, LTRI or severe LRTI). There is broad agreement between the two models, but the SAI projects markedly greater impact for the effect of reducing SLRTI risk, while the BWI projected markedly greater impact for risk of infection and risk of URTI. Both models projected less impact of maternal vaccination when compared with infant vaccination. Reduction in hospitalizations for the majority of the TPPs for maternal vaccination evaluated were in the region of 7% (SAI model) and 15% (BWI model) (see the Supplementary file 3D).

The multi-way sensitivity analysis gave support to the results of the one-way sensitivity analysis, agreeing with the prediction that the most significant impact on hospitalization of children under 5 years of age would be achieved using a vaccine design with reduced duration of infection and infectiousness. The multi-way analysis also agreed on the significance of duration of immunity, compliance and coverage on the predicted impacts. Finally, the multi-way analysis affirmed that there was no significant difference in the impact of the vaccine given different assumptions about the vaccine interactions with the maternal and naturally acquired immunity (see the Supplementary file 3A and 3B), or assumptions on the dose and various vaccination schedules (see the Supplementary file 3C). Overall the predictions for infant vaccination are strengthened by the fact that similar results arise from two models with different epidemiological structure. Investigations so far suggest uncertainty of the impact of a maternal vaccine, where both models showed relatively different impact of this type of vaccination when compared with the infant vaccinations.

## 4 Discussion

We report on an investigation of the potential population level impact of a range of vaccination strategy options, using an array of vaccine properties and vaccination schedules called TPPs, on disease arising from RSV using two transmission dynamic models.

In agreement, both models showed the maximum reduction of RSV hospitalization obtained from the vaccine effects on shortening duration and lowering infectiousness of RSV infection. This “herd protection effect” could most likely be explained by a significant fall in transmission in the risk group in which there was the strongly age-specific risk of severe disease coupled with age-homophilic (assortative) mixing patterns. The importance of indirect protection in the control of RSV is in accord with earlier published findings [4]. This could be related to the age specific contact pattern and that may be setting/country specific, thus it would be worthwhile to repeat the analysis in different settings.

The study provides a degree of robustness not frequently used in modelling evaluations of the potential impact of vaccination by undertaking a consensus analysis involving two models [6]. The findings from the two models are qualitatively similar and in some areas in quantitative agreement. Furthermore, what is unusual in this study is the emphasis placed on evaluating the properties associated with the vaccine in order to provide information that is of use in the process of vaccine development. For example, we show by sensitivity analysis that two key areas to focus on in vaccine design is the influence of the vaccine on reducing the duration and infectiousness of challenge infection subsequent to vaccination. These are in part altruistic properties of the vaccine insofar as they work by increasing the indirect benefits of vaccination to the unvaccinated.

In conclusion, the key elements of a most effective vaccine or vaccine strategy are (i) as expected, longer duration of protection and higher coverage, but also (ii) reductions in the duration of infection and degree of infectiousness are strongly influential. The lower incremental benefit from a vaccine reducing the risk of primary infection appears at first paradoxical. However, the scenarios chosen were of relatively low effect (up to 50%). The vaccine impact appears also insensitive to the possible interactions between the vaccine and existing immunity, for both acquired and passive antibodies.

## 5 Summary of the key findings

(1) The impact projected by the two models is qualitatively similar across the range of TPPs.
(2) The TPP options with highest predicted impacts offer significant reduction in hospitalizations of children under 5 years of age (around 70%).
(3) Vaccine characteristics that reduce the duration and infectiousness of infections in vaccinees are projected to have the greatest impact on hospitalized RSV. This is a consequence of a strong herd immunity effect of the vaccine programme.
(4) The impact of vaccination targeted at pregnant women is found to be model dependent, with minimal impact predicted for one model compared with the other. This is an interesting area for further research.

## Conflicts of interest

TVE was an employee of the GSK group of companies at the time this study was carried out and he is now currently an employee of Janssen R&D. ST and JFT are employees of the GSK group of companies. ST, JFT and TVE own shares of the GSK group of companies.

## Contributions

WP, TK, DJN, LJW – the conception/model design, and drafting the article. MK, ST, JFT, TVE – revising it critically for important intellectual content. WP, TK, SS – constructing and running mass simulations. All authors – final approval of the version to be submitted.

## Figures and Tables

**Table 1:** Vaccine effects – low, medium and high. The bold figures represent the baseline values used when comparing different TPPs.

Effect^a^	Low	Medium	High	Note and references
(i) Risk of primary infection reduction	**0%**	25%	50%	Respiratory vaccines tend not to generate sterilizing immunity in humans. Some pre-clinical data obtained with mucosally-administered viral-vectored RSV vaccine candidates suggest that the sterilizing immunity (no evidence of infection) of 50% can be achieved [14]
(ii) Duration of infectivity reduction	0%	**50%**	75%	Evidence from the RSV vaccines in pre-clinical models [14], [15] and from several influenza vaccines in animal models and human [16], [17], [18]. Antiviral candidates targeting RSV F protein mediated-fusion [19], a mechanism also targeted by RSV protein F-based vaccines, have been shown to reduce the level and duration of shedding in human challenge studies [20]
(iii) Infectiousness reduction	0%	**50%**	75%	
(iv) Risk of URTI reduction	**0%**	50%	75%	Influenza vaccines offer relatively modest efficacy against milder (URTI) disease but greater protection against more severe disease forms [21]. For RSV, preclinical model suggested that ten-fold higher neutralizing antibody levels are required to offer protection against upper, compared to lower, respiratory tract infection [22]. A 70% or higher efficacy against severe RSV disease seems a reachable goal considering the 78% efficacy observed with the monoclonal preparation Palivizumab in premature infants without bronchopulmonary dysplasia [23]
(v) Risk of LRTI reduction	50%	**70%**	90%	
(vi) Risk of severe LRTI (SLRTI) reduction	50%	**70%**	90%	

a Relative to the infected state in unvaccinated individuals.
