# Supplementary material for: Predicting the relative impacts of maternal and neonatal respiratory syncytial virus (RSV) vaccine target product profiles: A consensus modelling approach
Source: Vaccine. 2017 Jan 5;35(2):403–9. doi: 10.1016/j.vaccine.2016.10.073 (PMC5221409; doi:10.1016/j.vaccine.2016.10.073)
Supplement: Supplementary File 1 [file mmc1.pdf]

Supplementary 1: Models and Simulations

Demographic sub-model

In the SAI model, the demographic sub-model was divided into 99 age classes comprising 24 monthly age classes in the first two years of life and yearly age classes from the third year of life to age 75 years. The final age class is composed of individuals older than 75 years. The BWI model had only 15 age classes consisting of 12 monthly age classes in the first year of life, 2-5 year olds, 6-10 year olds and the final one composed of individuals aged 10 – 75 years old. The selection of monthly age classes in the youngest individuals is chosen so as to capture the transmission dynamics and the impact of vaccination in age groups who are the most at risk of RSV-related disease. Realistic population structure projections are obtained by use of age-specific mortality and fertility rates from KHDSS [1]. However, these rates are fixed in the model at the 2007 schedules, since to incorporate year on year changes would make it difficult to tell whether features projected by the model with vaccination are a result of demographic, epidemiological or vaccination patterns. The number of people in each age class is allowed to vary as a result of a continuous ageing process from a younger to an older age group and through natural deaths. The rate of ageing is taken to be the reciprocal of the length of the source age class. Assuming that an age class i can be represented as $[a_i, a_{i+1}]$, then the rate of ageing from age class i to i + 1 is thus as $\frac{1}{a_{i+1} - a_i}$.

Seasonal forcing

In the absence of a definitive knowledge of the drivers of RSV seasonal patterns, seasonality is modelled using a simple cosinusoidal function. The age-specific force of infection is expressed as:

$$\lambda_i(t) = \sum_{j=1}^{n}\left(\frac{\beta_{i,j}(1+a\cos(2\pi(t-\varphi)))}{N_j(t)}\sum_k \alpha_{k,j} I_{k,j}(t)\right)$$

where $\beta_{i,j}$ represents the WAIFW matrix (the transmission coefficient between susceptible of age class *i* and infected of age class *j*), $N_j(t)$ is the total number of infected individuals in age class *j* at time t, $\alpha_{k,j}$ is the relative infectiousness of infected individuals in different infected classes *k (k* = 0,1 and 2 in SAI model and *k* = asymptomatic (A), upper respiratory tract infection (URTI), lower respiratory tract infection (LRTI), severe lower respiratory tract infection (SLRTI) and hospitalization (H) in BWI model – see below and the supplementary Materials). The seasonal parameters defining the relative amplitude, *a*, and the timing of the peak in transmission, φ, are unknown and are determined by fitting the model to hospitalization data (see detail in *Model fitting and simulation*).

Disease stages and severity

The distribution of disease in those infected was assumed to be dependent upon the prior immune status. The distribution follows the decision tree shown in Figure 1, such that disease of increasing severity is always a subset of the preceding disease severity group. Based on a longitudinal cohort study of RSV infection in Kilifi, Kenya [2], the age-specific risk of a specific disease state for each individual following infection is estimated by fitting the static models to the data set. The initial vector used for the fitting process was derived from longitudinal data of RSV infections [3]. Here, static model refers to the equilibrium state with force of infection that is age-specific but constant in time. Once estimated from the static model, the risk of hospitalization remains fixed for fitting the dynamic models.

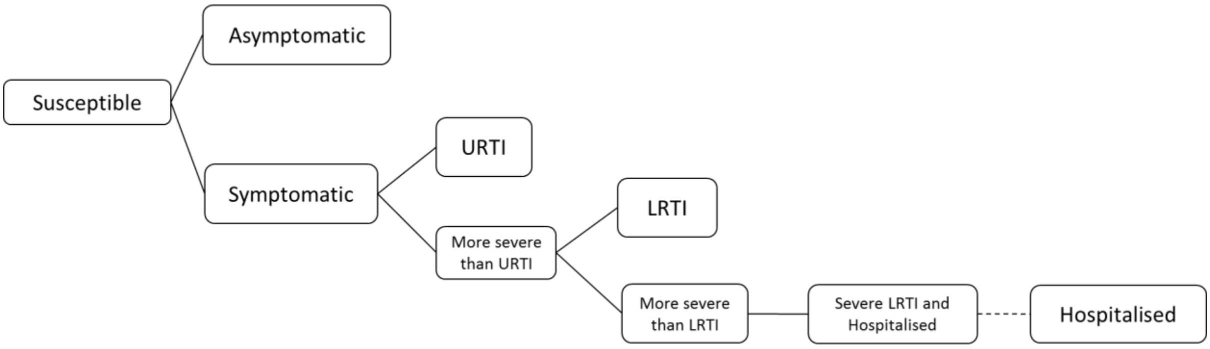

Figure 1.1S. Decision tree for distribution of disease states following infection. Solid lines represent the nested compartments within each other sequentially and the dashed line represents a proportion of severe LRTI who are hospitalized.

Two independent epidemiological sub-models

a) *Sequential acquisition of immunity model (SAI)*

This model is based on the epidemiological premise that following primary, secondary and subsequent infections the individual progressively develops an immunity state (with altered susceptibility to, and infectivity on, reinfection), which does not wane. The overall compartmental structure is defined in the Figure 1A in the main text and is described by Kinyanjui *et. al.* 2015 [4].

The host population is stratified into ten epidemiological groups: those who are maternally protected (M), primary susceptible (S_0), primary infected (I_0), primary recovered (P_0), secondary susceptible (S_1), secondary infected (I_1), secondary recovered (P_1), tertiary susceptible (S_2), tertiary infected (I_2) and tertiary recovered (P_2). Disease arising from infection is defined as D and hospitalization is defined as H. The incidence of disease (D) is related to age and episode while hospitalization (H) is related to age. All the state variables (i.e. the rectangle compartments in Figure 1.1S) are stratified by both age and time such that $S_{0,a}(t)$ represents the density of the primary susceptible of age *a* at time t and so forth. The rates, with respect to both time and age, at which individuals flow from one epidemiological

state to another are described by a system of ordinary differential equations shown below, where κ is the rate of ageing, Θ is the age-specific fertility rate, γ_0, γ_1 and γ_2 are the recovery rates from primary, secondary and tertiary infections respectively, ω is the rate of loss of maternal antibody protection, ρ_1 and ρ_2 are the rates of loss of secondary and tertiary immunity respectively, λ_a is the age-specific force of infection, N_a is the total number of people in age class a and μ_a is the age-specific mortality rate.

SAI model equations are as follow:

$$\frac{dM_{a,q}}{dt} = \begin{bmatrix} \sum_{\forall a} \Theta_a N_a \, if \; a = 1 \\ 0 if \; a \geq 2 \end{bmatrix} - M_{a,q}(\mu_a + \omega_a p_M) - \kappa_a M_{a,q} \, if \; q = 1$$

$$\frac{dM_{a,q}}{dt} = \kappa_{a-1} M_{a-1,q} + M_{a,q-1} \omega_a p_M - M_{a,q}(\mu_a + \omega_a p_M) - \kappa_a M_{a,q} \, if \; 2 \leq q \leq p_M$$

$$\frac{dS_{0,a}}{dt} = \kappa_{a-1} S_{0,a-1} + \omega_a p_M M_{a,p_M} - S_{0,a}(\mu_a + \lambda_a) - \kappa_a S_{0,a}$$

$$\frac{dI_{0,a}}{dt} = \kappa_{a-1} I_{0,a-1} + \lambda_a S_{0,a} - I_{0,a}(\mu_a + \gamma_{0,a}) - \kappa_a I_{0,a}$$

$$\frac{dP_{0,a}}{dt} = \kappa_{a-1} P_{0,a-1} + \gamma_{0,a} I_{0,a} - P_{0,a}(\mu_a + \rho_{0,a}) - \kappa_a P_{0,a}$$

$$\frac{dS_{1,a}}{dt} = \kappa_{a-1} S_{1,a-1} + \rho_{0,a} P_{0,a} - S_{1,a}(\mu_a + \sigma_{1,a} \lambda_a) - \kappa_a S_{1,a}$$

$$\frac{dI_{1,a}}{dt} = \kappa_{a-1} I_{1,a-1} + \sigma_{1,a} \lambda_a S_{1,a} - I_{1,a}(\mu_a + \gamma_{1.a}) - \kappa_a I_{1,a}$$

$$\frac{dP_{1,a}}{dt} = \kappa_{a-1} P_{1,a-1} + \gamma_{1,a} I_{1,a} - P_{1,a}(\mu_a + \rho_{1,a}) - \kappa_a P_{1,a}$$

$$\frac{dS_{2,a}}{dt} = \kappa_{a-1} S_{2,a-1} + \rho_{1,a} P_{1,a} + \rho_{2,a} P_{2,a} - S_{2,a}(\mu_a + \sigma_{2,a} \lambda_a) - \kappa_a S_{2,a}$$

$$\frac{dI_{2,a}}{dt} = \kappa_{a-1} I_{2,a-1} + \sigma_{2,a} \lambda_a S_{2,a} - I_{2,a}(\mu_a + \gamma_{2,a}) - \kappa_a I_{2,a}$$

$$\frac{dP_{2,a}}{dt} = \kappa_{a-1} P_{2,a-1} + \gamma_{2,a} I_{2,a} - P_{2,a}(\mu_a + \rho_{2,a}) - \kappa_a P_{2,a}$$

$$where : \lambda_a(t) = \sum_{j=1}^{n} \left(\frac{\beta_{a,j}(1 + \xi \cos(2\pi(t - \phi)))}{N_a(t)} \sum_{k=0}^{2} \alpha_k I_{k,j}(t) \right)$$

b) Boosting and waning of immunity model (BWI)

The BWI model has its origins in a simple non-age-structured model of RSV group A and B dynamics [5], but is essentially developed *de novo* as an age-stratified model for the purpose of evaluating the potential impact of vaccination. In the BWI model the host population is stratified into seven epidemiological compartments shown in Figure 1B in the main text: maternally protected (M), primary susceptible (S_0), infected but asymptomatic (A), infected and symptomatic categorized as upper respiratory tract infections (URTI), lower respiratory tract infections (LRTI), severe lower respiratory tract infections (SLRTI), or hospitalized (H), and, finally, secondary susceptible (S_1), that is, those still susceptible to infection, but who have partial immunity. The proportions of individuals entering each class upon infection are dependent on both age and immune status. The diagram shows the flow of individuals through the epidemiological compartments. The infection classes i.e. SLRTI, LRTI, URTI, and A are nested within each other sequentially (see Figure1 and decision state section above.) This excludes H which is not a separate infection class but a proportion of the SLRTI class. Similar to the SAI model, the model includes age-dependent processes, such as the force of infection and age-specific parameters. Thus all the state variables are stratified by both age and time such that $S_{0,i}(t)$ represents the density of the primary susceptible of age i at time t and so forth. The rates, with respect to both time and age, at which individuals flow from one epidemiological state to another are described a system of ordinary differential equations.

BWI model equations are as follow:

$$\frac{dM_1}{dt} = dM_1 + birth * N$$

$$\frac{dM_a}{dt} = \kappa_{a-1}M_{a-1} - \omega_m M_a - \mu_a M_a - \kappa_a M_a$$

$$\frac{dS_{0,a}}{dt} = \kappa_{a-1}S_{0,a-1} + \omega_m M_a + \rho S_{1,a} - S_{0,a}(\mu_a + \lambda_{0,a}) - \kappa_a S_{0,a}$$

$$\frac{dI_{A,a}}{dt} = \kappa_{a-1}I_{A,a-1} + \lambda_{0,a}(1 - SYMPTOM_{0,a})S_{0,a} + \lambda_{1,a}(1 - SYMPTOM_{1,a})S_{1,a} - I_{A,a}(\mu_a + \gamma_A) - \kappa_a I_{A,a}$$

$$\frac{dI_{U,a}}{dt} = \kappa_{a-1}I_{U,a-1} + \lambda_{0,a}SYMPTOM_{0,a}(1 - LRTI_{0,a})S_{0,a} + \lambda_{1,a}SYMPTOM_{1,a}(1 - LRTI_{1,a})S_{1,a} - I_{U,a}(\mu_a + \gamma_U) - \kappa_a I_{U,a}$$

$$\frac{dI_{L,a}}{dt} = \kappa_{a-1}I_{L,a-1} + \lambda_{0,a}SYMPTOM_{0,a}LRTI_{0,a}(1 - SEVERE_{0,a})S_{0,a} + \lambda_{1,a}SYMPTOM_{1,a}LRTI_{1,a}(1 - SEVERE_{0,a})S_{1,a} - I_{L,a}(\mu_a + \gamma_L) - \kappa_a I_{L,a}$$

$$\frac{dI_{SL,a}}{dt} = \kappa_{a-1}I_{SL,a-1} + \lambda_{0,a}SYMPTOM_{0,a}LRTI_{0,a}SEVERE_{0,a}S_{0,a} + \lambda_{1,a}SYMPTOM_{1,a}LRTI_{1,a}SEVERE_{1,a}S_{1,a} - I_{SL,a}(\mu_a + \gamma_{SL}) - \kappa_a I_{SL,a}$$

$$\frac{dS_{1,a}}{dt} = \kappa_{a-1}S_{1,a-1} + I_{A,a}\gamma_A + I_{U,a}\gamma_U + I_{L,a}\gamma_L + I_{SL,a}\gamma_{SL} - S_{1,a}(\mu_a + \lambda_{1,a} + \rho) - \kappa_a S_{1,a}$$

where $\lambda_{0,a}(t) = \sum_{j=1}^{n}(\frac{\beta_{a,j}(1+\xi\cos(2\pi(t-\phi)))}{N_a(t)}\sum_{k=\{A,U,L,SL,H\}}\alpha_k I_{k,j}(t))$

$$\lambda_{1,a}(t) = \sum_{j=1}^{n}(\frac{(1-\sigma)\beta_{a,j}(1+\xi\cos(2\pi(t-\phi)))}{N_a(t)}\sum_{k=\{A,U,L,SL,H\}}\alpha_k I_{k,j}(t))$$

and $N = M + S_0 + I_A + I_U + I_L + I_{SL} + S_1 A$

There are two main differences between the structure of the BWI model and the structure of the SAI model: First, there is an absence of any fully resistant to infection state for the BWI model (compare with the short lived fully immune classes P_0, P_1, and P_2 in the SAI model). Instead, individuals move directly into the secondary susceptible class S_1 after their infection. In this class, people are less likely to get infected and if they are infected, they are less likely to get severe infection. Second, in the BWI model, immunity can wane and secondary susceptible individuals in S_1 could return to completely naïve susceptible (S_0) i.e. if an individual remains unchallenged for a certain length of time, they can return to S_0. For simplicity, tertiary and subsequent infections were all classified as being secondary infections in the BWI model.

Note that in both the SAI and BWI model RSV related deaths are assumed sufficiently few not to impact on population and infection dynamics, i.e. negligible for the modelling purpose, and hence they are not explicitly represented in the model equations.

Model fitting and simulation

Both models were implemented as ordinary differential equations (ODEs) and solved numerically (for SAI model, an explicit Runge-Kutta method of order (4,5) with an adaptive time step was used in Matlab1 [6], for BWI model the "`deSolve`" package of R v3.2.1 with "`lsoda`" solver was used [7]). The two models were calibrated using the same set of age- and time-specific hospitalization data obtained from long-term surveillance of pneumonia admissions to Kilifi County Hospital, coastal Kenya [8]. The model was fitted using statistical maximum-likelihood estimation assuming that the age and time counts follow a Poisson distribution. We optimized the model parameters by maximizing the log-likelihood as

$$\sum_{a=1}^{n}\left\{\sum_{t=1}^{T_a}\left(k(a,t)\log H(a,t) - H(a,t) - \sum_{j=1}^{k(a,t)}\log(j)\right)\right\}$$

where k(a,t) is the expected incidence of hospitalizations at age a and time t, T_a is the number of time points at which the expected incidence data are made for each age a and H(a,t) is the corresponding expected incidences from the model at each age class a and time t. n is the number of age classes used in each model.

To ensure that the impacts of each vaccine TPP would not be influenced by transient population and infection dynamics, the initial conditions for the state variables for each age class were taken to be the pre-vaccination numbers obtained by running the model for a period of 100 years to its stable limit cycle. Model projections with vaccination were over a ten year time horizon and stratified into age group (infants aged under one year and individuals less than five years of age), for (a) a baseline epidemiological and TPP parameter set, and (b) a wide range of TPP sets forming the main sensitivity analysis.

Sensitivity analyses

We approach the sensitivity analysis in two stages, one-way and multi-way analyses.

> Stage 1: A one-way sensitivity analysis cycling through the vaccine TPP parameters and varying each parameter sequentially through each option defined above while keeping the others at baseline. This leads to 26 simulations and the results are described and discussed here. This analysis was done twice, one for infant and the other for maternal vaccination regimens.
>
> Stage 2: A multi-way sensitivity analysis on the 6 vaccine effect parameters defined in Table 1 of the main text (every combination of 3 values for each of the 6 effect parameters i.e. $3^6 = 729$ combinations), combined with a one-way sensitivity analysis of all the other parameters (i.e. a process as for stage 1 including all the vaccine implementing strategies, waning and possible interactions with immunities) with an attempt to identify the key vaccine characteristics that correlate with the level of vaccine impact on the key outcome, i.e. severe disease in infancy (specifically hospitalizations due to RSV in the first 12 months of life). This will be 729×13=9477 simulations. This process is undertaken only for infant vaccination.

References

[1] Scott JA, Bauni E, Moisi JC, Ojal J, Gatakaa H, Nyundo C, et al. Profile: The Kilifi Health and Demographic Surveillance System (KHDSS). Int J Epidemiol. 2012;41:650-7.

[2] Ohuma EO, Okiro EA, Ochola R, Sande CJ, Cane PA, Medley GF, et al. The natural history of respiratory syncytial virus in a birth cohort: the influence of age and previous infection on reinfection and disease. Am J Epidemiol. 2012;176:794-802.

[3] Nokes DJ, Okiro EA, Ngama M, Ochola R, White LJ, Scott PD, et al. Respiratory syncytial virus infection and disease in infants and young children observed from birth in Kilifi District, Kenya. Clinical infectious diseases : an official publication of the Infectious Diseases Society of America. 2008;46:50-7.
[4] Kinyanjui TM, House TA, Kiti MC, Cane PA, Nokes DJ, Medley GF. Vaccine Induced Herd Immunity for Control of Respiratory Syncytial Virus Disease in a Low-Income Country Setting. PloS one. 2015;10:e0138018.
[5] White LJ, Waris M, Cane PA, Nokes DJ, Medley GF. The transmission dynamics of groups A and B human respiratory syncytial virus (hRSV) in England & Wales and Finland: seasonality and cross-protection. Epidemiology and infection. 2005;133:279-89.
[6] IncM. MATLAB. 7.7.0 ed. US-MA-Natick, Mathworks; 2014.
[7] Soetaert K. PT, Setzer R.W. Solving differential equations in R: Package deSolve. J Stat Softw. 2010;33:25.
[8] Nokes DJ, Ngama M, Bett A, Abwao J, Munywoki P, English M, et al. Incidence and severity of respiratory syncytial virus pneumonia in rural Kenyan children identified through hospital surveillance. Clinical infectious diseases : an official publication of the Infectious Diseases Society of America. 2009;49:1341-9.
