# Supplementary material for: Predicting the relative impacts of maternal and neonatal respiratory syncytial virus (RSV) vaccine target product profiles: A consensus modelling approach
Source: Vaccine. 2017 Jan 5;35(2):403–9. doi: 10.1016/j.vaccine.2016.10.073 (PMC5221409; doi:10.1016/j.vaccine.2016.10.073)
Supplement: Supplementary File 2 [file mmc2.doc]

# Supplementary 2: Model parameters for SAI and BWI model

| **Parameter symbol** | **Description** | **SAI model**  **mean (95%CI)** | **BWI model**  **mean (95%CI)** | | **Data source** |
| --- | --- | --- | --- | --- | --- |
| Seasonality parameters | | | | | |
| $\xi$ | Amplitude | 0.10 (0.09-0.1) | 0.24 (0.21 - 0.26) | |  |
| $\varphi$ | Phase angle | 2.86e-06 (-0.008-0.008) | 9.63e-04 | |  |
| Transmission parameters | | | | | |
| $q$ | Infectivity parameter | 0.00222 (0.00216-0.0023) | 0.091 (0.085 - 0.098) | |  |
| $\gamma$ | Rate of recovery from primary infection, $I_{0}$ (SAI) or A, URTI (BWI) per year | $\gamma_{1},\gamma_{2}$=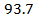 (Fixed) | 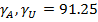 (Fixed) | |  |
|  | Rate of recovery from secondary and subsequent infections: $I_{1}, I_{2}$ (for SAI) or LRTI, SLRTI (for BWI) per year | $\gamma_{0}$=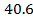 (Fixed) | 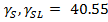 (Fixed) | |  |
| Immunity parameters | | | | | |
| $\omega$ | Duration of RSV specific maternal antibody protection (in months) | 2.03 (1.91 – 2.69) | 0.31(0.12- 0.76) |  | |
| $\sigma$ | Immunity factor reducing the susceptibility of previously exposed individuals in $S_{1}$ (both) and $S_{2}$ (SAI only) | $\sigma_{1}=0.75$(Fixed)  $\sigma_{2}=0.65$(Fixed) | $\sigma=0.54$ |  | |
| $\rho$ | Rate of waning of short-term immunity of recovered individuals: $P_{k}:k=0,1,2$(SAI) per year | $\rho_{0},\rho_{1},\rho_{2}=2$(Fixed) | 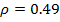 |  | |
| $\alpha$ | Factor reducing infectiousness of reinfected individuals $I_{1}$ and $I_{2}$ (SAI) or SLRTI, LRTI, URTI, A (BWI) | $\begin{matrix} \alpha_{1}=0.5 \\ \alpha_{2}=0.25 \end{matrix}$ (Fixed) | 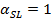 (Fixed)  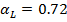 (0.70 -0.74)  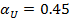(0.43-0.48)  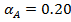 (0.17 -0.22) |  | |

Table 2.1S. Parameter estimates for SAI and BWI models

The result of optimization of both models against the same data set (time- and age-structured hospitalization data for Kilifi, coastal Kenya) have identified two important features of the models. First, the difference in the force of infection where in the BWI, this value was lower than in the SAI model. Secondly, the different estimate of the duration of maternal antibody protection, this value was much smaller in the BWI model compared to the SAI model estimate (1 week versus 2 months). These two results are related insofar as the shorter duration of maternal protection is necessary in order to allow sufficient early age infection (and hence disease) despite the force of infection being lower (or despite the average age at primary infection is higher for BWI than SAI). Given a lower force of infection (higher age at first infection) and shorter maternal immunity then the impact of infant vaccination projected (and discussed in the next section) is greater for BWI than for SAI since the vaccination window is wider.

**References**

[1] Hall C, Geiman J, Biggar R, Kotok D, Hogan P, Douglas RJ. Respiratory syncytial virus infections within families. New England Journal of medicine. 1976;294:414-9.

[2] Okiro EA, White LJ, Ngama M, Cane PA, Medley GF, Nokes DJ. Duration of shedding of respiratory syncytial virus in a community study of Kenyan children. BMC Infect Dis. 2012;10:15.

[3] Waris M, Meurman O, Mufson MA, Ruuskanen O, Halonen P. Shedding of infectious virus and virus antigen during acute infection with respiratory syncytial virus. J Med Virol. 1992;38:111-6.

[4] Henderson F, Collier A, Clyde WJ, Denny F. Respiratory-syncytial-virus infections, reinfections and immunity. A prospective, longitudinal study in young children. New England Journal of Medicine. 1979;300:530-4.

[5] Agoti CN, Mwihuri AG, Sande CJ, Onyango CO, Medley GF, Cane PA, et al. Genetic relatedness of infecting and reinfecting respiratory syncytial virus strains identified in a birth cohort from rural Kenya. The Journal of infectious diseases. 2012;206:1532-41.

[6] Ohuma EO, Okiro EA, Ochola R, Sande CJ, Cane PA, Medley GF, et al. The natural history of respiratory syncytial virus in a birth cohort: the influence of age and previous infection on reinfection and disease. Am J Epidemiol. 2012;176:794-802.

[7] Scott PD, Ochola R, Ngama M, Okiro EA, James Nokes D, Medley GF, et al. Molecular analysis of respiratory syncytial virus reinfections in infants from coastal Kenya. The Journal of infectious diseases. 2006;193:59-67.
