# Supplementary material for: Predicting the relative impacts of maternal and neonatal respiratory syncytial virus (RSV) vaccine target product profiles: A consensus modelling approach
Source: Vaccine. 2017 Jan 5;35(2):403–9. doi: 10.1016/j.vaccine.2016.10.073 (PMC5221409; doi:10.1016/j.vaccine.2016.10.073)
Supplement: Supplementary File 3 [file mmc3.pdf]

Supplementary 3: Model uncertainties and other related data sets

A) Vaccine and maternal antibodies

A range of scenarios are plausible for the potential interaction of an infant vaccine in the presence of a maternal antibody presence that is waning. Three options explored within this study were:

i) "No interaction" (baseline choice) Interaction between vaccine-induced immunity and natural immunity i.e. if the vaccine dose is administered in the presence of maternal antibodies the vaccine has the same effect as in the absence of maternal antibodies

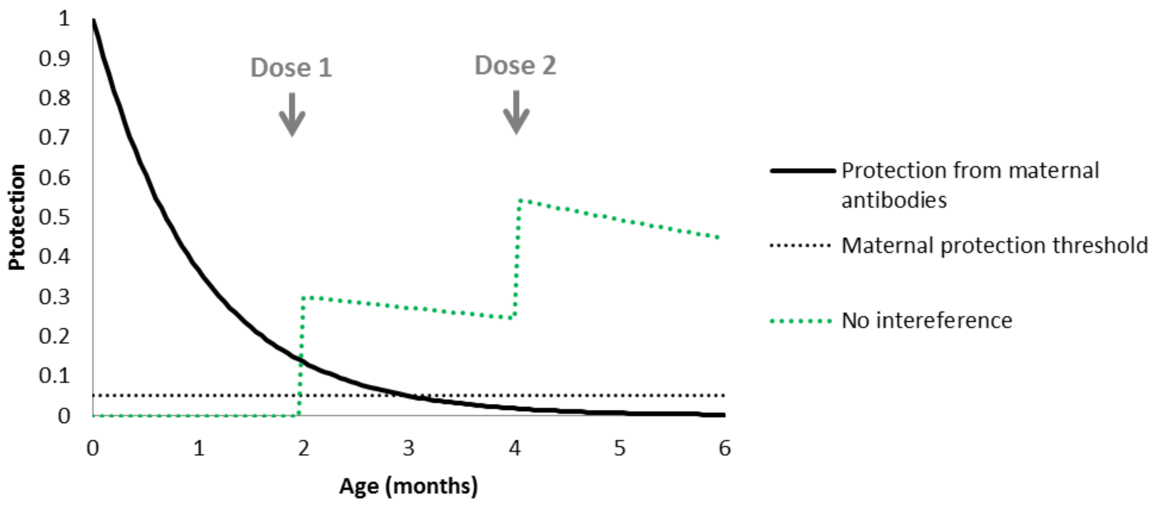

Figure 3.1S. No interaction between vaccine and maternal antibodies

ii) “Bounce up” i.e. if the first dose(s) is given in the presence of maternal antibodies, then it will have no effect, and then if the subsequent doses are in the absence of maternal antibodies, vaccine protection would have the same effect as in those who received the same number of doses in the absence of maternal antibodies.

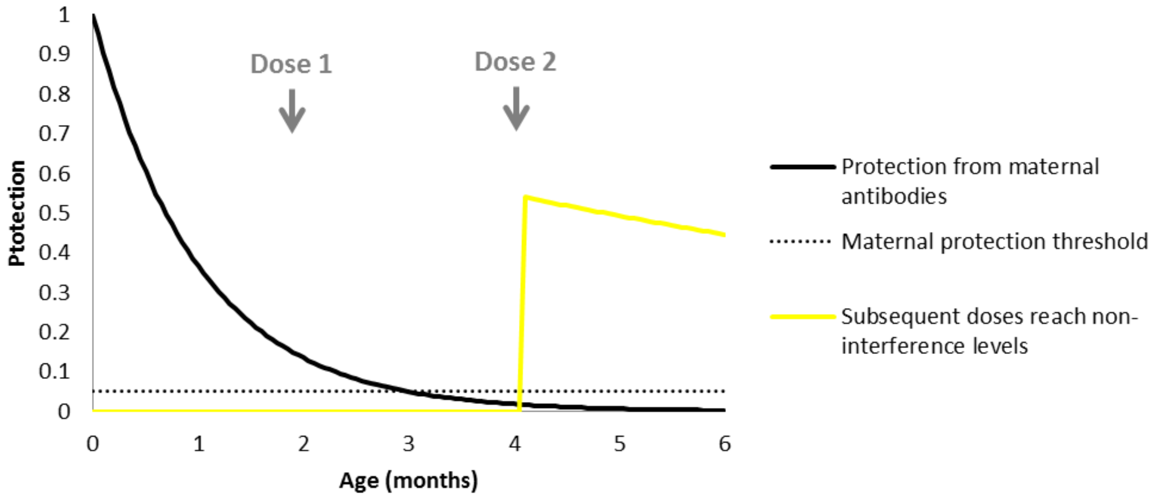

Figure 3.2S. Bounce up effect of vaccine in the absence of maternal antibodies

iii) “Drop back” i.e. if the first dose is given in the presence of maternal antibodies it will have no effect and then if the second is given in the absence of maternal antibodies it will have the same effect as a first dose in the absence of maternal antibodies and the third dose would have the effect of a second dose. If the first and second doses are given in the presence of maternal antibodies they will have no effect and then if the third dose is given in the absence of maternal antibodies then it would have the effect of a first dose.

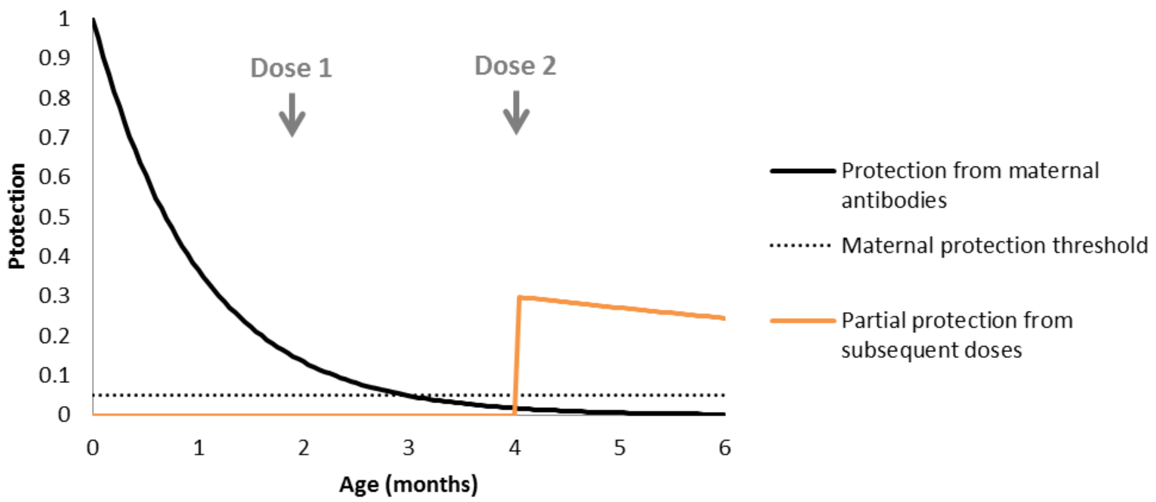

Figure 3.3S. Drop back effect of vaccine in the absence of maternal antibodies

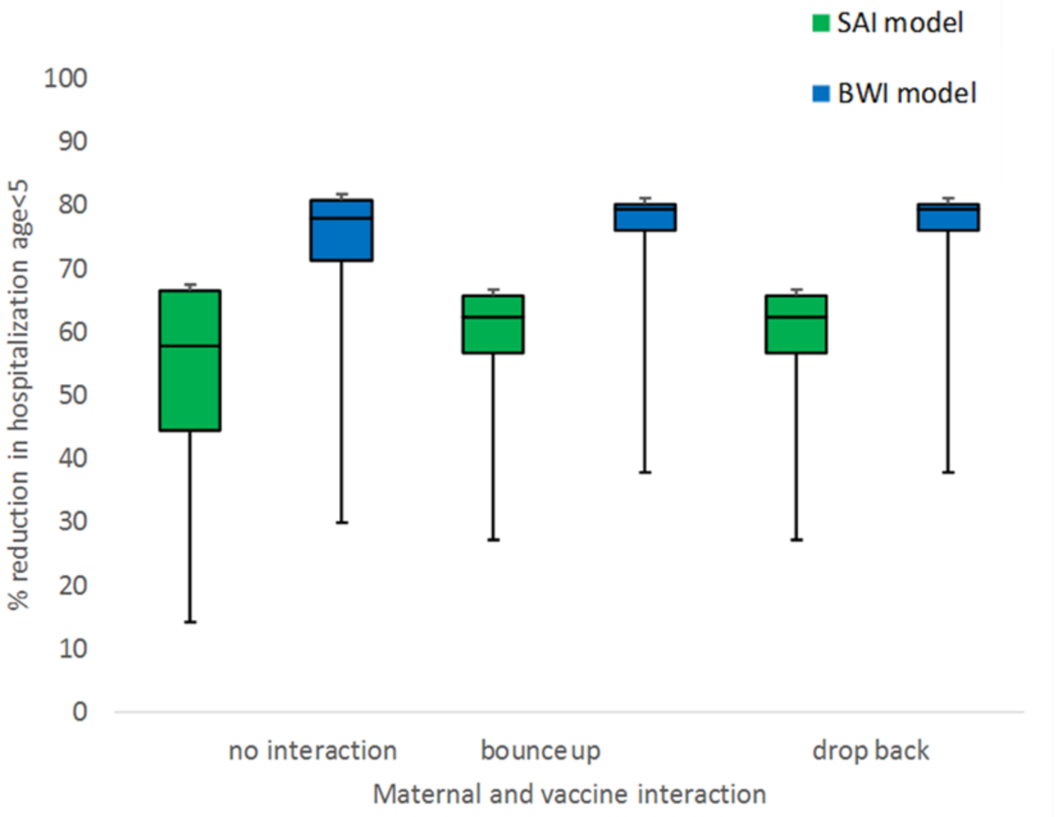

Figure 3.4S. Comparison of impact of three forms of interaction between an infant vaccine in the presence of maternal immunity, for the SAI model (green) and the BWI model (blue).

The percentage reduction in hospitalization of under 5 year olds predicted to arise from vaccination, stratified by three possible scenarios for interaction of vaccines with maternal immunity, i.e. no interaction, bounce up and drop back, for the two model structures. For all three interaction types explored in the study, both SAI and BWI models show little impact of different kinds of interaction between maternal antibodies and vaccine impact.

Regarding the uncertainty of the underlying biology, other kinds of interactions include “Permenant Inteference”, “Revert to non-interference levels below threshold” and “Boosting” are possible. These options have clear implications for the choice of dosing regimen and TPP of the vaccine.

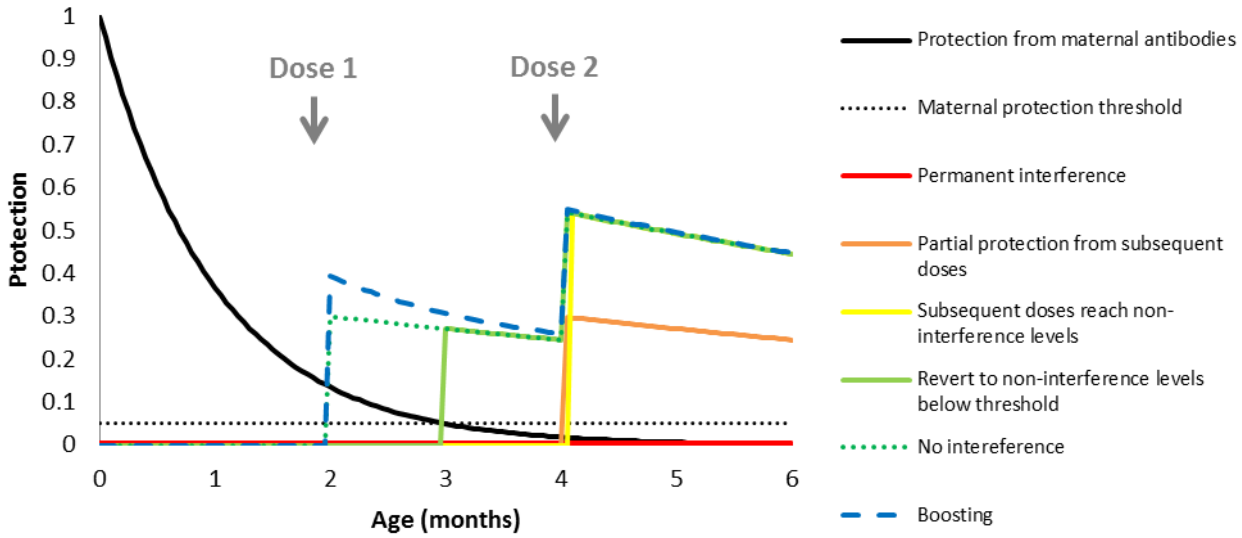

Figure 3.5S. Different types of interaction between vaccine and maternal antibodies

B) Vaccine and Natural induced immunities

Three options of effects of vaccination in individuals with natural immunity were explored

i) "No effect" (baseline choice) i.e. if the vaccine is given to individuals with some natural immunity it will have no effect, which would be in keeping with results of trials of RSV live attenuated vaccines

ii) "Multiplicative" i.e. if the vaccine is given to individuals with some natural immunity it will have a combined effect with the protection provided by the natural immunity and

iii) "Top-up" i.e. if the vaccine is given to individuals with some natural immunity the resulting protection would be the maximum of the vaccine-induced and natural immunity.

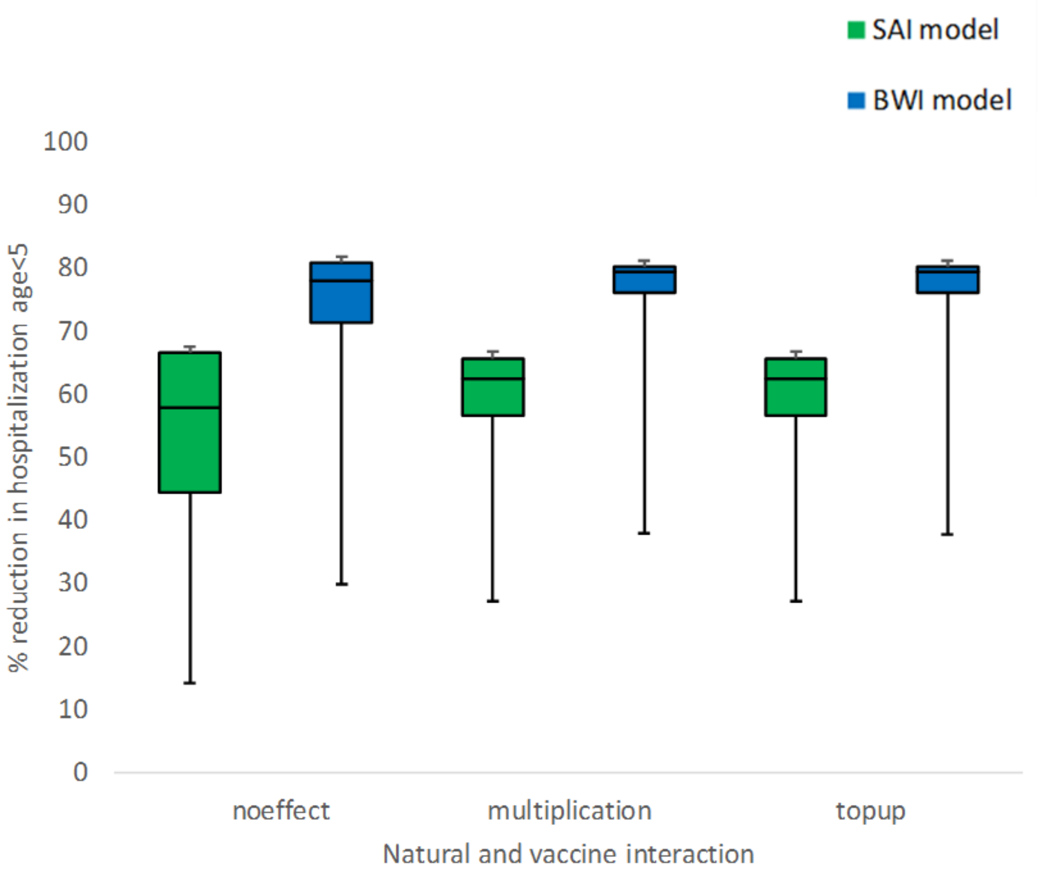

Figure 3.6S: Comparison of impact of three forms of interaction between an infant vaccine in the presence of natural immunity, for the SAI model (green) and the BWI model (blue).

The percentage reduction in hospitalization of under 5 year olds predicted to arise from vaccination, stratified by three possible scenarios for interaction of vaccines with natural immunity, i.e. no interaction, multiplicative and top up, for both model structures. Interaction with natural immunity assumed to inhibit the effect of the vaccine leads to lower impact. However, both SAI and BWI models show little impact of such interactions on the percentage of hospitalization of children ages less than 5 years old.

C) Dosing schedule and vaccine impacts

Infant vaccination was assumed to be either through 2 or 3 doses and at various ages of delivery as follow:

i) 2 doses at 0 and 2 months of age

ii) 2 doses at 2 and 4 months of age (baseline choice)

iii) 3 doses at 0, 1 and 2 months of age

iv) 3 doses at 2, 4 and 6 months of age

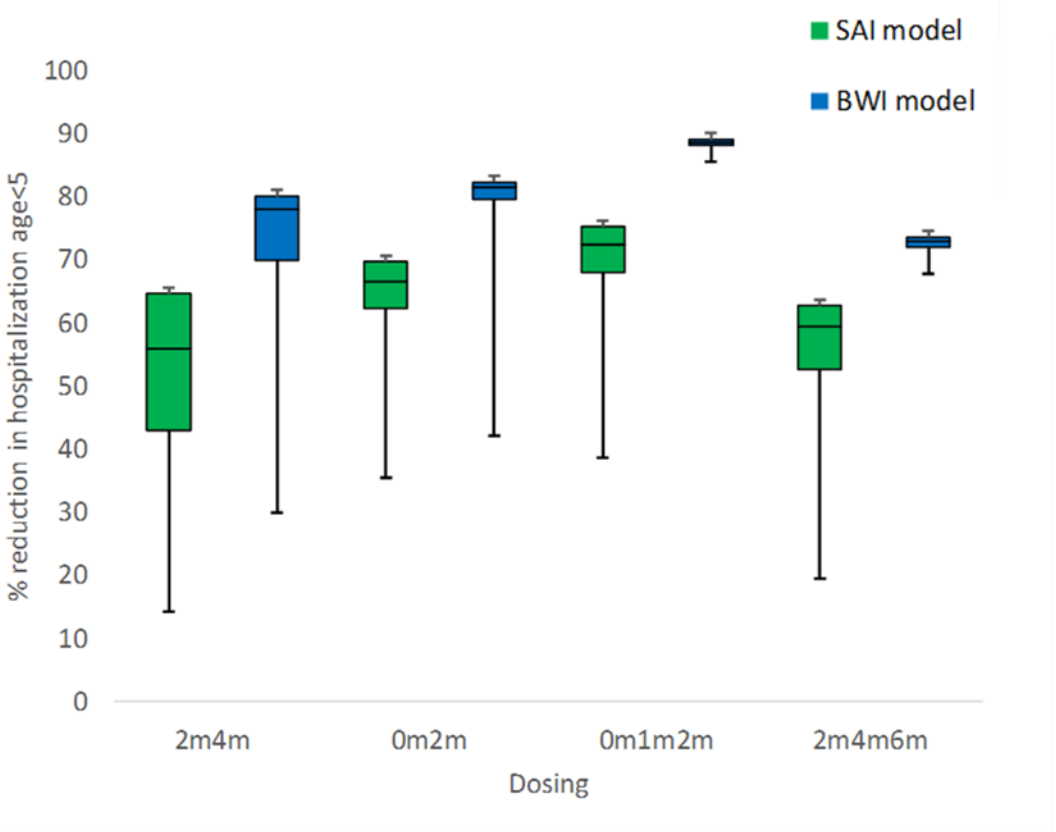

Figure 3.7S: Comparison of impact of four dosing schedules of an infant vaccine.

Box and whisker plots to show the percentage reduction in hospitalization of under 5 year olds predicted to arise from vaccination, stratified by four different dosing schedules, for the SAI model (green) and the BWI model (blue).

The key result is that although the two models differ to some degree (greater impact of BMI relative to SAI), from the multi-way sensitivity analysis, both agreed that the three doses given at 0, 1 and 2 months was the optimal regimen.

D) Maternal vaccination

a. b.

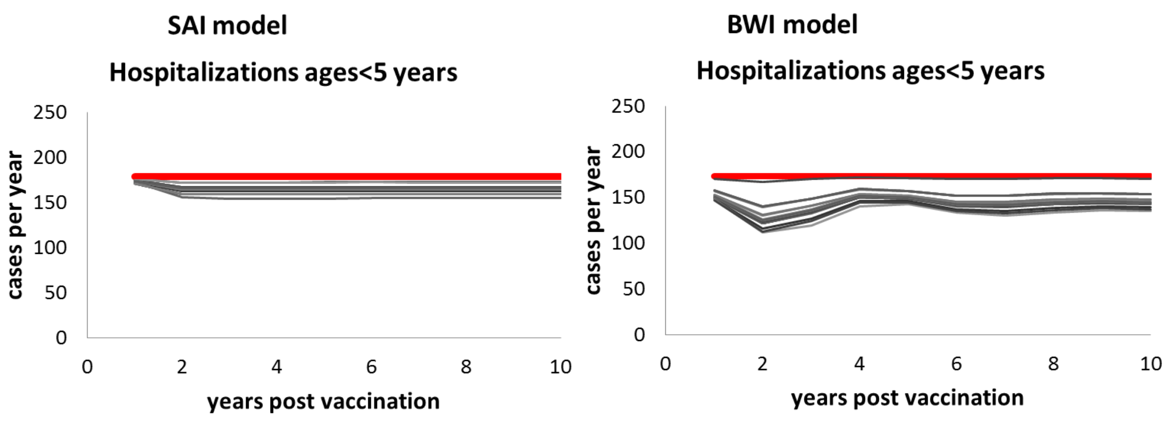

Figure 3.8S: Comparison of predictions of two models of the impact of maternal vaccination a) the SAI model b) the BWI model predictions of impact of vaccine TPPs on hospitalizations of children under 5 years old over time since vaccination begins. Each graph plots the non-vaccine model fit (bold solid red line) and the 24 TPPs of the one-way sensitivity analysis (grey lines).

Maternal vaccination was predicted to have a reduced impact compared with infant vaccination with most TPPs predicted to lead to a reduction in hospitalizations in the region of 7% (SAI model) and 15% (BWI model). Both models predict that impact can be improved by increased duration of vaccine effect and increased coverage. Interaction with natural immunity which works to inhibit the effect of the vaccine leads to lower impact. The other TPPs considered are predicted to have fairly similar impacts to each other by each model.

E) Other related data sets for the modelling

a) Population structure of Kilifi Health and Demographic Surveillance System (KHDSS)

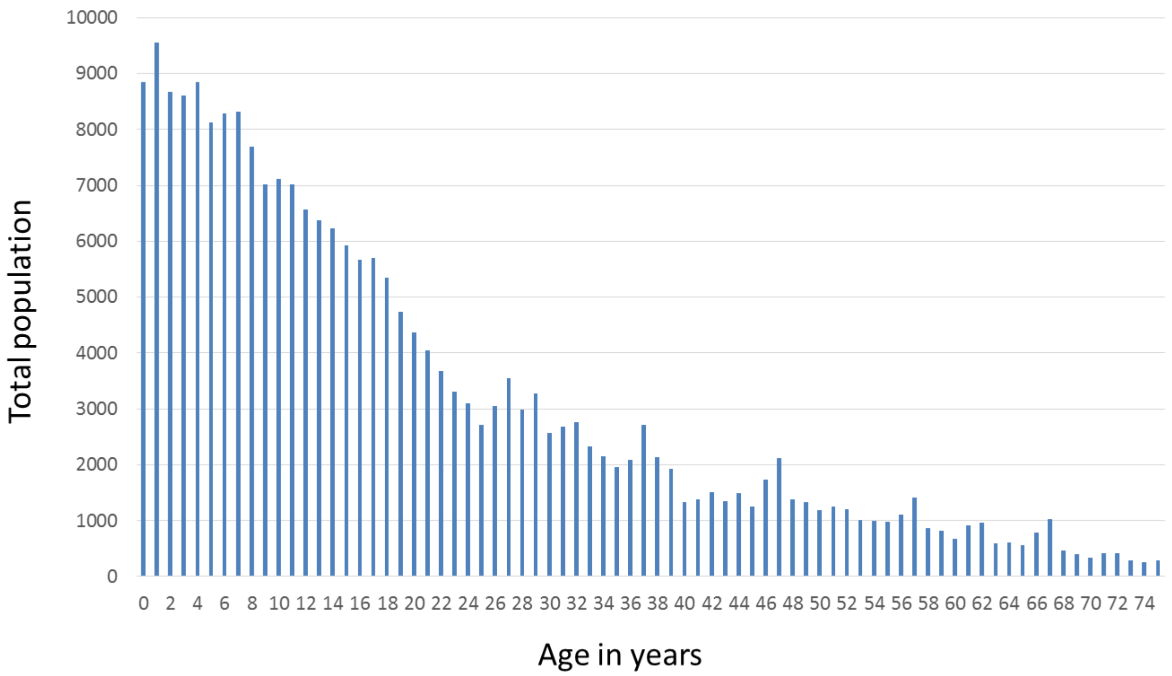

Figure 3.9S: Population structure of Kilifi Health and Demographic Surveillance System (KHDSS) in 2007

b) Kenya age-specific contact rates

To collect the social contacts data, we conducted a study in five locations in the northern part of the KHDSS [1] spanning a rural to semi-urban transect. Participants from within 6 age groups were randomly chosen from the KHDSS enumeration registers with numbers in proportion to each location size. Age categories reflected key social and behavioral characteristics; <1 (infants), 1-5 (pre-school), 6-14 (primary school), 15-19 (secondary school), 20-49 (adults) and >50 years (fully mature) years. Each participant was expected to keep the diary for a single, randomly chosen day of the week (between the time of waking up and going to bed) and for each person they contacted, record their age class

and the frequency of encounters. A contact was defined as a skin-to-skin contact (e.g. hugging, shaking hands and kissing). From this study, we evaluated the average number of physical contacts that individuals in age class i make with individuals in age class j per day, then corrected for the age specific proportion of population who participated in the survey i.e. sampling weights, this is then denoted by C_{ij}.

We used the social contact data to construct the WAIFW matrix, denoted as β_{ij}, which was then used in the models to estimate the force of infection (FOI), that is, per susceptible rate of infection. We assume that the age-specific number of potentially infectious contacts is proportional to the self-reported age-specific number of social contacts, i.e. $\beta_{ij} = qC_{ij}$, where the proportionality constant, q, is a proportionality factor that measures the disease specific infectivity and estimated by fitting the model to age-specific hospitalization data [2].

	Contact age group (yrs)						
Participant's age group (yrs)		<1	1-5	6-15	16-19	20-49	>50
	<1	0.2 (0.1-0.3)	2.7 (2.3-3.2)	4.6 (4.0-5.4)	1.3 (1.1-1.7)	4.0 (3.4-4.7)	1.0 (0.7-1.2)
	1-5	0.5 (0.4-0.7)	4.4 (3.8-5.2)	6.0 (5.1-6.9)	1.5 (1.2-1.8)	4.1 (3.5-4.7)	1.1 (0.9-1.4)
	6-15	0.6 (0.4-0.7)	3.8 (3.2-4.4)	8.9 (7.9-10.1)	2.3 (1.9-2.7)	3.6 (3.1-4.2)	0.9 (0.7-1.1)
	16-19	0.5 (0.3-0.7)	2.0 (1.6-2.5)	5.5 (4.6-6.4)	5.2 (4.4-6.1)	5.0 (4.2-5.8)	1.1 (0.9-1.4)
	20-49	0.7 (0.5-0.8)	2.5 (2.1-2.9)	3.1 (2.7-3.6)	2.1 (1.8-2.5)	8.2 (7.3-9.3)	2.3 (1.9-2.6)
	>50	0.4 (0.2-0.6)	1.5 (1.1-2.0)	2.5 (1.9-3.1)	1.4 (1.0-1.9)	6.0 (4.8-7.4)	2.1 (1.6-2.7)

Table 3.1S. Age group specific contact rate per day per participant for Kilifi, Kenya (with 95% CI). From a diary study conducted in Kilifi, Kenya 2010. Confidence interval (CI) based on 2000 bootstraps

References

[1] Kiti MC, Kinyanjui TM, Koech DC, Munywoki PK, Medley GF, Nokes DJ. Quantifying age-related rates of social contact using diaries in a rural coastal population of Kenya. PloS one. 2014;9:e104786.

[2] Kinyanjui TM, House TA, Kiti MC, Cane PA, Nokes DJ, Medley GF. Vaccine Induced Herd Immunity for Control of Respiratory Syncytial Virus Disease in a Low-Income Country Setting. PloS one. 2015;10:e0138018.
